# Asymptomatic hyperuricemia and coronary flow reserve in patients with metabolic syndrome

**DOI:** 10.1186/s41927-018-0027-6

**Published:** 2018-06-20

**Authors:** Seoyoung C. Kim, Marcelo F. Di Carli, Rajesh K. Garg, Kathleen Vanni, Penny Wang, Alyssa Wohlfahrt, Zhi Yu, Fengxin Lu, Anarosa Campos, Courtney F. Bibbo, Stacy Smith, Daniel H. Solomon

**Affiliations:** 1 Division of Pharmacoepidemiology and Pharmacoeconomics, Department of Medicine, Brigham and Women’s Hospital, Harvard Medical School, 1620 Tremont St, Suite 3030, Boston, MA 02120 USA; 2 Division of Rheumatology, Immunology, and Allergy, Department of Medicine, Brigham and Women’s Hospital, Harvard Medical School, Boston, MA USA; 3 Division of Nuclear Medicine, Department of Radiology, Brigham and Women’s Hospital, Harvard Medical School, Boston, MA USA; 4 Division of Cardiovascular Medicine, Department of Medicine, Brigham and Women’s Hospital, Harvard Medical School, Boston, MA USA; 5 Division of Endocrinology, Diabetes & Hypertension, Department of Medicine, Brigham and Women’s Hospital, Harvard Medical School, Boston, MA USA; 6 Department of Radiology, Brigham and Women’s Hospital, Harvard Medical School, Boston, MA USA

**Keywords:** Uric acid, Metabolic syndrome, PET/CT, DECT, Coronary blood flow

## Abstract

**Background:**

Patients with metabolic syndrome (MetS) are at increased risk of asymptomatic hyperuricemia (i.e., elevated serum uric acid (SUA) level without gout) and cardiovascular disease. We conducted a cross-sectional study to examine associations between SUA levels and coronary flow reserve and urate deposits in carotid arteries in patients with asymptomatic hyperuricemia and MetS.

**Methods:**

Adults aged ≥40 years with MetS and SUA levels ≥6.5 mg/dl, but no gout, were eligible. Using a stress myocardial perfusion positron emission tomography (PET), we assessed myocardial blood flow (MBF) at rest and stress and calculated coronary flow reserve (CFR). CFR < 2.0 is considered abnormal and associated with increased cardiovascular risk. We also measured insulin resistance by homeostatic model assessment (HOMA-IR) method and urate deposits using dual-energy CT (DECT) of the neck for the carotid arteries.

**Results:**

Forty-four patients with the median age of 63.5 years underwent a blood test, cardiac PET and neck DECT scans. Median (IQR) SUA was 7.8 (7.1–8.4) mg/dL. The median (IQR) CFR was abnormally low at 1.9 (1.7–2.4) and the median (IQR) stress MBF was 1.7 (1.3–2.2) ml/min/g. None had urate deposits in the carotid arteries detected by DECT. In multivariable linear regression analyses, SUA had no association with CFR (β = − 0.12, *p* = 0.78) or stress MBF (β = − 0.52, *p* = 0.28). Among non-diabetic patients (*n* = 25), SUA was not associated with HOMA-IR (β = 2.08, *p* = 0.10).

**Conclusions:**

Among MetS patients with asymptomatic hyperuricemia, we found no relationship between SUA and CFR, stress MBF, and insulin resistance. No patients had any DECT detectable subclinical urate deposition in the carotid arteries.

## Background

The association between hyperuricemia, with and without gout, and risk of coronary artery disease (CAD), metabolic syndrome and kidney disease has been well-reported [1–9]. However, debate persists as to whether serum uric acid (SUA) has a causal role in the development of these conditions. Metabolic syndrome or diabetes is a known risk factor for CAD as results of macro- and micro-angiopathy related to diabetes [10, 11]. Patients with both metabolic syndrome and hyperuricemia may be at increased cardiovascular risk.

Positron emission tomography (PET)-measured coronary flow reserve (CFR) - the ratio of peak hyperemic myocardial blood flow (MBF) over that at rest as– is shown to be a reliable imaging marker of clinical cardiovascular risk [12, 13]. A reduced CFR can be a sign of flow-limiting CAD [14] and presence of coronary vascular dysfunction involving smaller vessels, which increases the severity of inducible myocardial ischemia and sub-clinical myocardial injury beyond the effects of upstream coronary obstruction [15]. CFR less than 2.0 has been shown to be independently associated with risk for CAD, heart failure as well as cardiovascular death [12, 13, 16, 17]. While the association between gout, hyperuricemia and cardiovascular disease has been extensively studied, it has not been studied whether asymptomatic hyperuricemia (i.e., hyperuricemia without known diagnosis of gout) is associated with coronary vascular function measured with PET-CFR.

Dual-energy computed tomography (DECT) is a highly specific imaging modality that allows specific detection and volume measurement of urate crystals in the joints or tendons among patients with tophaceous gout [18, 19]. In a recent meta-analysis of 8 studies on DECT diagnostic performance, the pooled sensitivity was 84.7% and the pooled specificity 93.7% for gout [20]. DECT also had the positive predictive value of 87% for diagnosing gout in patients with a history of gout during their intercritical period [21]. While in some studies up to 24% had DECT-positive urate deposits in the joints of asymptomatic hyperuricemic patients [22, 23], no data is available whether urate crystals exist and/or can be detected in the vasculature using DECT scans.

We, therefore, conducted a cross-sectional study to determine the association between SUA levels and CFR, insulin resistance, renal function, and systemic inflammation. In addition, we used DECT scans to examine whether we could find/visualize subclinical urate deposits in carotid arteries among patients with asymptomatic hyperuricemia and metabolic syndrome.

## Methods

### Study population

For this cross-sectional study, eligible patients were men and women aged 40 years or older who had asymptomatic hyperuricemia defined as SUA ≥6.5 mg/dL and metabolic syndrome defined by the presence of at least 3 out of 5 traits in the National Cholesterol Education Program – Adult Treatment Panel III (NCEP-ATP III) criteria [i.e., obesity with body mass index (BMI) > 29.4 kg/m^2^, high triglyceride level, low high-density lipoprotein level, hypertension, or hyperglycemia] [24]. We excluded pregnant or nursing women, patients with diagnosis of gout, symptomatic coronary artery disease or pulmonary disease, moderate-to-severe valvular heart disease requiring surgery, end-stage renal disease, renal replacement therapy, active malignancy requiring treatment, or those who used xanthine oxidase inhibitors, colchicine or probenecid. Details of this study cohort is described elsewhere [25].

The study protocol was approved by the Institutional Review Board of the Brigham and Women’s Hospital. Written informed consent was obtained in all included patients before participating the study.

### Patient recruitment

We recruited patients from the Partners Healthcare Biobank (https://biobank.partners.org) or several clinical sites of the Brigham and Women’s Hospital (BWH). After we identified potential patients who met the study criteria through medical record review, we contacted those patients via letter. All patients went through a structured pre-screen phone call or a visit. We measured the SUA level by enzymatic colorimetric assay at the screening visit, unless a SUA value ≥6.5 mg/dL from within the last year was available in their medical record.

### Positron emission tomographic imaging

Patients underwent a whole-body PET/computed tomography scanner (Discovery RX or STE LightSpeed 64, GE Healthcare, Milwaukee, WI) after at least 4 h of fasting. The study protocol for PET is similar to our previous work described elsewhere [26]. Briefly, ^13^N-ammonia was used as a flow tracer at rest and stress for PET, [27] and an intravenous infusion of regadenoson was given as a stressor. We quantified MBF in ml/min/g during rest and peak stress using ^13^N-ammonia and calculated CFR as the ratio of stress MBF over rest MBF [28–31]. Clinically relevant cardiologic variables including heart rate, blood pressure, and 12-lead ECG were assessed at baseline and throughout the test. With commercially available software, we calculated left ventricular ejection fraction (LVEF) at rest and stress from gated myocardial perfusion images. In addition, summed rest, stress, and difference scores were computed. Higher summed stress scores reflect larger areas of myocardial scar and ischemia. In general, normal scans have the summed stress score ≤ 3 [32–34].

### Dual-energy CT (DECT) imaging

We obtained DECT scans of the neck using a dual-source CT scanner operated at DECT mode (SOMATOM Definition Flash, Siemens Medical Systems, Forchheim, Germany) at the tube potentials of 80 kV and 140 kV with an additional tin filter. We then used a commercial software post-processing program (‘Gout’, Syngo CT Workplace, Siemens Medical Systems) to produce digital color-coded images, where MSU deposits were marked as green. As a part of the main study, the study patients also underwent a DECT scan of the foot described elsewhere [25].

### Markers of systemic inflammation and metabolic risks

We measured markers of systemic inflammation including interleukin (IL)-6 and high-sensitivity C-reactive protein (hs-CRP), and markers of metabolic risks including lipid, insulin and glucose levels at fasting. IL-6 level was assessed by enzyme-linked immunosorbent assay (ELISA). Insulin level was measured using a 2-site electrochemiluminescent immunoassay on the Roche automated platform. We then quantified insulin resistance using the Homeostatic Model Assessment-Insulin Resistance (HOMA-IR, normal < 3) method [35]. We also collected information on a number of predefined variables potentially related to hyperuricemia or cardiometabolic risk, including demographics, body mass index (BMI), smoking status, comorbidities, and medication use. In addition, we measured serum creatinine and urine microalbumin and estimated glomerular filtration rate (eGFR) for the kidney function.

### Statistical analysis

We used descriptive statistics to characterize the study cohort. Because data were not normally distributed, we used natural log transformation of SUA levels, CFR, MBF, and other laboratory results as dependent variables in regression models. For the primary analysis, we used unadjusted and multivariable linear regression models to examine the association between SUA levels and coronary vascular function (i.e., CFR and stress MBF) in the main cohort. Our final models were adjusted for age, sex, BMI, summed stress score (i.e., a strong indicator of myocardial scar and ischemia), serum creatinine, IL-6, hs-CRP, and presence of diabetes. Because prior myocardial scar or ischemia is a major determinant of CFR, we conducted a sensitivity analysis in which we performed multivariable linear regression models only in patients with summed stress scores which measure the extent of myocardial scar and ischemia ≤3 [32–34]. For the association between SUA and HOMA-IR, we ran unadjusted and multivariable linear regression in a subgroup of patients without diabetes.

Because no patients had subclinical urate deposits in the neck DECT scan, no further analysis was done for that variable. We used SAS 9.4 Statistical Software (SAS Institute Inc., Cary, NC) for all analyses.

## Results

**Fig. 1:**
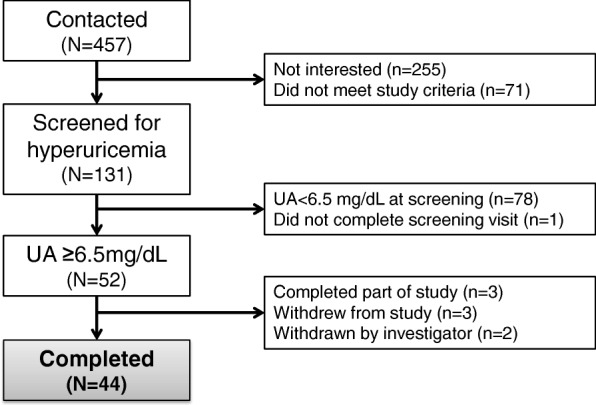
Patient recruitment flow chart. Among 457 patients we contacted, 52 (11.4%) patients had hyperuricemia defined as having serum uric acid (UA) ≥6.5 mg/dL, and 44 (9.6%) completed the study

A total of 131 patients were consented into the study. Of these, 78 (59.5%) were excluded because of absence of hyperuricemia. One patient did not complete the screening blood draw. Eight patients who had hyperuricemia did not complete the full study; three declined to participate further, three patients completed only a portion of the study, and two were withdrawn by the study investigator. Forty-four completed the full study (see Fig. 1). Median age (IQR) was 65 (64–67) years, median (IQR) SUA was 5.5 (5.0–6.1) mg/dL and 66.7% were male in 86 patients who were consented but did not complete the study visit. Among those who completed the study, median [Interquartile range (IQR)] age was 63.5 (58.0–68.5) years, median (IQR) SUA was 7.8 (7.1–8.4) mg/dL and 40.9% were male (Table 1). The median (IQR) BMI was 34.7 (32.0–41.8) kg/m^2^ and 43.2% had type 2 diabetes. Half of patients had a family history of MI and 11.4% had a history of MI.

**Table 1:** Study patient characteristics

Total number of patients	44
Demographic	
Age, year, median (IQR)	63.5 (58.0–68.5)
Male, n (%)	18 (40.9)
Comorbidities	
Body mass index, kg/m^2^, median (IQR)	34.7 (32.0–41.8)
Current smoking, n (%)	3 (6.8%)
Type 2 diabetes, n (%)	19 (43.2%)
Insulin use, n (%)	6 (13.6%)
MI, n (%)	5 (11.4%)
Statin use, n (%)	33 (75.0%)
Family history of MI, n (%)	22 (50.0%)
10-year Reynolds risk score, %, median (IQR)	11.2 (4.2–19.4)
Laboratory data*,* median (IQR)	
Uric acid, mg/dL	7.8 (7.1–8.4)
Total cholesterol, mg/dL	167.5 (153.0–198.0)
Triglycerides, mg/dL	172.5 (115.0–201.5)
HDL, mg/dL	44.0 (38.0–54.0)
LDL, mg/dL	87.0 (76.5–116.5)
Fasting blood glucose, mg/dL	100.5 (92.5–135.0)
Serum creatinine, mg/dL	0.9 (0.8–1.2)
eGFR, mL/min/1.73m^2^	48.5 (34.5–57.5)
Fasting insulin, mIU/L	18.2 (14.4–21.9)
HOMA-IR	4.8 (3.4–6.5)
hs-CRP, mg/L	2.9 (1.1–7.4)
Interleukin-6, pg/mL	4.5 (2.4–6.8)
Urine microalbumin, mg/L	15.0 (7.5–43.4)
Cardiovascular function*,* median (IQR)	
Systolic blood pressure, mmHg	131 (123–146)
Diastolic blood pressure, mmHg	65 (61–76)
Rest heart rate, per minute	72 (64–78)
Stress heart rate, per minute	94 (84–103)
Rest myocardial blood flow, mL/min/g	0.8 (0.7–0.9)
Stress myocardial blood flow, mL/min/g	1.7 (1.3–2.2)
Coronary flow reserve	1.9 (1.7–2.4)
Rest left ventricular ejection fraction, %	60.0 (52.0–67.0)
Stress left ventricular ejection fraction, %	63.0 (54.5–70.0)
Summed stress score	0 (0–6)
Summed rest score	0 (0–0)
Summed difference score	0 (0–5)

IQR = interquartile range, MI = myocardial infarction, eGFR = estimated glomerular filtration rate, HDL = high-density lipoprotein, LDL = low-density lipoprotein, HOMA-IR = Homeostatic Model Assessment of Insulin Resistance (normal < 3), hs-CRP = high sensitivity C-reactive protein

The median (IQR) CFR was 1.9 (1.7–2.4) and median (IQR) stress MBF was 1.7 (1.3–2.2) ml/min/g. Twenty-six (57.8%) patients had CFR less than 2.0 known to be associated with worse cardiovascular outcomes in a general referral population [16]. Twenty-eight (62.2%) had a normal summed stress score (≤3) which is a marker of prior myocardial scar or ischemia [32–34]. The median (IQR) HOMA-IR was 4.8 (3.4–6.5). In the unadjusted linear regression analyses (Table 2), SUA was not associated with coronary vascular function (CFR and stress MBF), systemic inflammation (IL-6 and hs-CRP), and insulin resistance (HOMA-IR). However, SUA had a positive association with serum creatinine (β = 0.87, *p* = 0.01) and an inverse association with eGFR (β = − 1.23, *p* = 0.002). In the final multivariable linear regression model adjusting for age, sex, diabetes, BMI, summed stress score, serum creatinine, IL-6 and hs-CRP (Table 3), SUA was not associated with CFR (β = − 0.12, *p* = 0.78) or stress MBF (β = − 0.52, *p* = 0.28).

**Table 2:** Unadjusted linear regression analysis for the association between serum uric acid and cardiometabolic function (*n* = 44)

Variables^a^	Standardized coefficient (SE)	*P*-value
Coronary flow reserve	0.04 (0.35)	0.90
Stress myocardial blood flow	−0.20 (0.43)	0.64
Interleukin-6	−0.46 (1.00)	0.65
Serum creatinine	0.87 (0.33)	0.01
HOMA-IR	0.76 (1.04)	0.47
hs-CRP	−1.47 (1.56)	0.35
eGFR	−1.23 (0.38)	0.002

^a^All the variables were log-transformed. SE = standard error, eGFR = estimated glomerular filtration rate, HOMA-IR = Homeostatic Model Assessment of Insulin Resistance, hs-CRP = high sensitivity C-reactive protein

**Table 3:** Multivariable linear regression analysis for the association between serum uric acid and cardiometabolic function

	Adjusted for	Standardized coefficient (SE)	P-value
All patients (n = 44)			
CFR	Age, sex	0.04 (0.35)	0.92
	Age, sex, diabetes, BMI, SSS, Cr	0.07 (0.39)	0.86
	Age, sex, diabetes, BMI, SSS, Cr, IL-6, and hs-CRP	−0.12 (0.42)	0.78
Stress MBF	Age, sex	−0.19 (0.40)	0.63
	Age, sex, diabetes, BMI, SSS, Cr	−0.35 (0.44)	0.43
	Age, sex, diabetes, BMI, SSS, Cr, IL-6, and hs-CRP	−0.52 (0.47)	0.28
Patients with summed stress score ≤ 3 (n = 28)			
CFR	Age, sex	0.17 (0.43)	0.69
	Age, sex, diabetes, BMI, SSS, Cr	0.21 (0.38)	0.60
	Age, sex, diabetes, BMI, SSS, Cr, IL-6, and hs-CRP	0.09 (0.43)	0.83
Stress MBF	Age, sex	−0.13 (0.42)	0.76
	Age, sex, diabetes, BMI, SSS, Cr	−0.13 (0.43)	0.76
	Age, sex, diabetes, BMI, SSS, Cr, IL-6, and hs-CRP	−0.23 (0.47)	0.63
Patients without diabetes (n = 25)			
HOMA-IR	Age, sex	1.87 (1.30)	0.17
	Age, sex, BMI	1.36 (1.26)	0.29
	Age, sex, BMI, IL-6 and hs-CRP	2.08 (1.21)	0.10

SE = standard error, CFR = coronary flow reserve, MBF = myocardial blood flow, BMI = body mass index, SSS = summed stress score, Cr = serum creatinine, IL = interleukin, hs-CRP = high sensitivity C-reactive protein**,** HOMA-IR = Homeostatic Model Assessment of Insulin Resistance

No association between SUA, CFR and stress MBF was noted in a sensitivity analysis limiting to 28 patients with a normal summed stress score (≤3). Among patients with no diabetes (*n* = 25), the median (IQR) HOMA-IR was 4.6 (3.8–5.7) and there was no significant association between SUA and HOMA-IR (β = 2.08, *p* = 0.1). None had DECT-detectable subclinical urate deposits in the neck, while 15% of these patients had subclinical urate deposits in the foot DECT scan (results published elsewhere) [25].

## Discussion

Over the past few decades, growing evidence from a number of large epidemiologic studies suggests that a higher SUA is independently associated with an increased risk of cardiovascular disease including CAD [2–9, 36, 37]. Elevated serum uric acid levels are thought to cause endothelial dysfunction via oxidative stress, micro-inflammation, lipid oxidation, and inhibition of nitric oxide production [38, 39]. However, the causality of such associations has not been proven [40, 41]. In this cross-sectional study of 44 patients with metabolic syndrome and asymptomatic hyperuricemia, 58% had abnormally low CFR (i.e., CFR < 2.0) known to be an independent predictor for worse cardiovascular risk [12, 13, 16, 17]. However, we found that SUA level was not associated with CFR, stress MBF, or HOMA-IR. Both unadjusted and adjusted analyses consistently yielded the null results. Due to the nature of the cross-sectional design, we were unable to determine an association between the duration of hyperuricemia and CFR.

There are several explanations for our null findings. First, it is possible that our study did not find any association between SUA and coronary vascular function or insulin resistance because our study was limited to those with hyperuricemia. Second, it is possible that hyperuricemia is not causally associated with coronary vascular function or insulin resistance in the absence of gout. Third, moderate hyperuricemia might not have a strong relationship with CFR even if SUA itself is causally related to cardiovascular risk. However, our results are consistent with another study of 382 patients with and without gout which showed no association between SUA level and CFR [26]. Fourth, since most patients in our study are older and have many other known strong cardiovascular risk factors such as obesity, hypertension, renal dysfunction, and diabetes, SUA may not have any additional effect on patients’ coronary vascular function even if it has a modest causal association with cardiometabolic risk. Third, this pilot study may be underpowered particularly at the level of moderately, not severely, high SUA. Fourth, since we did not have a normouricemic group to compare with, the difference in patients’ SUA levels might have been relatively too small.

A few prior studies examined the presence of subclinical urate deposits in patients with asymptomatic hyperuricemia using musculoskeletal ultrasound [42, 43]. DECT is a newer imaging modality that allows specific detection and volume measurement of urate crystals in the joints or tendons among patients with gout [18]. A validation study of DECT for gout showed a high specificity over 93% but a moderate sensitivity below 80% [19]. However, the sensitivity of DECT is noted to be low in non-tophaceous gout [44]. A few studies used DECT to assess subclinical urate deposits in patients with asymptomatic hyperuricemia. In a previous study of 25 patients with asymptomatic hyperuricemia (SUA ≥9.0 mg/dL), 24% were noted to have subclinical urate deposits in the joints and tendons based on the DECT scans of the feet [23]. In a cohort of renal transplant patients with asymptomatic hyperuricemia (*n* = 27, median SUA = 7.9 mg/dL), only 1 patient had quadriceps tendon deposition. However, none had articular or renal urate deposits [22]. In the present study, we also did not find any DECT-detectable urate deposits in the carotid arteries among hyperuricemic patients. It may be partially explained by the fact that most patients were hyperuricemic but their SUA were not too high with the upper quartile SUA level of 8.4 mg/dL. Furthermore, the sensitivity of DECT for the vasculature in asymptomatic hyperuricemia patient may be too low as 15% of the study cohort had DECT-positive urate deposits in their feet [25]. While it has been reported that urate deposits were present in the mitral valve, aortic and tricuspid valves and the endocardium in patients with gout, [45–47]. it remains unknown whether patients with asymptomatic hyperuricemia have urate deposits in the vasculature including the carotid arteries.

There are limitations in this study. First, this is a cross-sectional study without longitudinal followup. While we found no association between SUA and coronary vascular function and insulin resistance at one point in time, there could be an association between changes in SUA and changes in cardiometabolic risks. Second, since we included only asymptomatic hyperuricemic patients, the association between SUA and cardiometabolic risks may be different for patients with gout. Third, this study was performed at a single academic center and relied on active patient participation. Thus, the generalizability of our results may be limited. Patients who were enrolled but did not complete the study visit were older and more likely to be male and had lower SUA levels. Fourth, while this is one of the largest studies on asymptomatic hyperuricemia, the study size may not be adequate. In particular, only 25 patients (56.8%) had no diabetes. Thus, the subgroup analysis that included only non-diabetic patients on the association between SUA and HOMA-IR may be underpowered. Fifth, the final models were adjusted for several important predictors of cardiometabolic risk including age, sex, renal function, a summed stress score (i.e., a marker of myocardial scar and ischemia), and markers of systemic inflammation (i.e., IL-6 and hs-CRP), there may be residual confounding.

## Conclusions

In this cross-sectional study of patients with metabolic syndrome and asymptomatic hyperuricemia, we found no relationship between SUA, coronary vascular function, and other cardiometabolic markers. Further studies are needed to confirm our findings. None of the patients had DECT-detectable subclinical urate deposits in the neck.

## Abbreviations

BMI: body mass index
CAD: coronary artery disease
CFR: coronary flow reserve
CRP: c-reactive protein
DECT: dual-energy computed tomography
HOMA-IR: Homeostatic Model Assessment of Insulin Resistance
IL: interleukin
IQR: interquartile range
MBF: myocardial blood flow
MetS: metabolic syndrome
PET: positron emission tomography
SUA: serum uric acid
